# Multi-UAV Content Caching Strategy and Cooperative, Complementary Content Transmission Based on Coalition Formation Game

**DOI:** 10.3390/s22093123

**Published:** 2022-04-19

**Authors:** Yanzan Sun, Xinlin Zhong, Fan Wu, Xiaojing Chen, Shunqing Zhang, Nan Dong

**Affiliations:** 1 Key Laboratory of Specialty Fiber Optics and Optical Access Networks, Shanghai Institute for Advanced Communication and Data Science, Shanghai University, Shanghai 200444, China; yanzansun@shu.edu.cn (Y.S.); zhongxinlin@shu.edu.cn (X.Z.); jodiechen@shu.edu.cn (X.C.); shunqing@shu.edu.cn (S.Z.); 2 School of Economics, Shanghai University, Shanghai 200444, China; 3 Changan Automobile Software Technology Co., Ltd., Shanghai 201103, China; dongnan1@changan.com

**Keywords:** unmanned aerial vehicle (UAV), user clustering, caching, coalition formation game (CFG)

## Abstract

The transmission of a large amount of video and picture content brings more challenges to wireless communication networks. Unmanned aerial vehicle (UAV)-aided small cells with active content caching deployed on cellular networks are recognized as a promising way to alleviate wireless backhaul and support flexible coverage. However, a UAV cannot operate for a long time due to limited battery life, and its caching capacity is also limited. For this, a multi-UAV content-caching strategy and cooperative, complementary content transmission among UAVs are jointly studied in this paper. Firstly, a user-clustering-based caching strategy is designed, where user clustering is based on user similarity, concurrently taking into consideration similarities in content preference and location. Then, cooperative, complementary content transmission between multiple UAVs is modeled as a coalition formation game (CFG) to maximize the utility of the whole network. Finally, the effectiveness of the proposed algorithms is demonstrated through numerical simulations.

## 1. Introduction

With the vigorous development of wireless mobile networks, the popularization of various smart terminal devices and emerging applications has brought about a huge data storm. From 4G to 5G, global mobile data traffic is predicted to increase by 1000 times [1]. Current research shows that the main data source is the transmission of multimedia information, such as pictures and videos, to meet the social and entertainment requirements of smart terminals, where the repeated transmission of popular content accounts for most of the network load, which further causes increases of the backhaul-linked traffic burden and energy consumption of ground base stations [2].

Caching is considered a key implementation technology for content-centric cellular networks to reduce network traffic load [3]. It can be configured on the network devices at the edge of the wireless network, such as macro base stations (MBSs) and small base stations (SBSs), which store certain popular content from the core network in advance through a backhaul link with limited capacity. Then, the transmission distance of the content cached at the edge of the wireless network can be shortened, which effectively reduces transmission delay, alleviates network congestion, and, further, improves network energy efficiency and throughput [4,5].

At present, most existing caching is usually carried out on static networks without considering mobility, where the content is mostly cached in static ground base stations. However, for temporary hot events, such as sports activities or concerts, static ground base stations with cache may not be able to meet the temporary high-capacity requirements of users; further, the cost of building ground small base stations is also relatively high. Owing to UAVs’ agility and fast deployment, they can act as flying cache-enabled base stations that can adjust their locations flexibly, giving them natural advantages to provide higher network coverage and service for satisfying temporary high-capacity requirements of users [6]. Then, by deploying cache-enabled UAVs, the traffic load of ground BSs during peak hours of certain hotspots can be effectively alleviated with lower cost.

Most of the current research studies a single UAV caching strategy, and rarely considers cooperative transmission among UAVs. For the multi-UAV scenario serving temporary hot events, on the one hand, a UAV cannot operate for a long time due to its limited battery life, which will reduce the communication reliability. On the other hand, the caching capacity of one UAV is generally limited, which cannot efficiently satisfy users’ content requirement in an energy efficient way. Then, to efficiently design a content-caching strategy and realize cooperative, complementary content transmission among UAVs to improve network energy efficiency (EE), satisfy users and improve transmission reliability is still a challenging problem. To solve this problem, the contributions of this paper are summarized as two aspects:

- Firstly, a user-clustering method is proposed based on user similarity, which is jointly scaled by distance similarity and content preference similarity among users. Then, a user-clustering-based content caching strategy is proposed to optimize the kinds of content stored in the caching space of each UAV by jointly considering user preference and content popularity.
- Secondly, we model the multi-UAV cooperative, complementary transmission problem as a coalition-formation game and propose a cooperation-order-based coalition-formation-game (CO-CFG) algorithm to maximize the whole network utility, which is defined by jointly considering user satisfaction, transmission energy consumption of UAVs and cooperative, complementary transmission reliability of UAVs.

The remainder of this paper is organized as follows. The system model is described in Section 3. A user-clustering-based UAV caching strategy is proposed in Section 4. Multi-UAV cooperative, complementary transmission is investigated as a coalition-formation game in Section 5. Simulation experiments are carried out in Section 6, and finally, Section 7 concludes this paper.

## 2. Related Work

In traditional cellular networks, cache is mainly deployed to optimize network throughput [7,8], energy consumption [9,10], energy efficiency [11], transmission delay [12,13] and network utility [14,15]. Specifically, combining caching with resource management has been investigated to improve network throughput for device-to-device (D2D) wireless caching network in [7,8]. Minimization of energy consumption was studied from the point of view of an SBS sleeping mechanism, cell clustering and caching in [9,10]. One more step forward, EE was optimized by active caching optimization and users’ spatial redivision in [11]. Transmission delay reduction is also one of the advantages of caching. Content download delay and transmission costs were minimized by cooperative caching optimization among BSs in [12]. The average content delivery delay was reduced by caching placement strategy optimization for D2D-assisted HetNets in [13]. In order to improve the network utility, a utility-maximization problem with joint preference-aware cache deployment and cache space allocation was proposed for the D2D cache network in [14]. By using a non-cooperative game model, the SBS caching space was auctioned to content providers for placing content in [15]. Several edge-caching projects were made using the concept of cloudlets to leverage the geographical proximity of resources to mobile users and offer them a better user experience in [16,17,18]. In particular, a centralized, cloudlet-based architecture was investigated to reduce latency and facilitate access to data stored in the cloud by mobile users in [16]. A fundamental problem of service caching from remote data centers to edge cloudlets in a multi-tiered edge cloud network was studied for satisfying the users different service requirements in [17]. The authors in [18] proposed a novel analytical model of transient dynamics of the cloudlets set to improve system convergence, stability and to deliver content locality. Furthermore, autonomous content discovery and dissemination within high-density, low-mobility crowds was developed to enable users to retrieve content by themselves in [19].

The above research mainly focused on static and fixed networks with caching. However, in the case of temporary hotspots events, when local traffic increases, the cost of deploying small base stations is too high. Therefore, UAVs with flexible deployment and high mobility are studied for computing and caching. For example, a scenario of UAVs serving users as edge computing servers was studied in [20]. For UAV caching, the problem of actively deploying UAVs with caching capabilities to minimize transmission power was studied in [21]. High-speed, UAV-assisted secure transmission in an ultra-high-density network was investigated in [22], where video-stream caching using UAVs and SBSs at the same time was developed. Furthermore, several works have jointly considered UAV deployment and caching [23,24,25,26]. The authors studied the optimization of joint UAV deployment, caching placement and user association to achieve maximum quality of experience (QoE) in [23]. In [24], the authors proposed a joint UAV trajectory and proactive caching strategy to reduce content transmission delay. In [25], the file caching policy, UAV trajectory and communication scheduling were jointly optimized to minimize the weighted sum of file caching cost and retrieval cost. In [26], the placement of content caching and UAV location were optimized to maximize the throughput among internet of thing (IoT) devices.

A few attempts have been made for a multi-UAV collaborative network with caching. For content distribution and caching, ref. [27] proposed a novel distributed framework, which promoted selective file caching and collaborative file sharing to minimize the delay of delivering content. A context-based group-buying mechanism was proposed to reduce data cost in [28], where the group buying mechanism was modeled as a coalition-formation game (CFG). In [29], based on a CFG, a collaborative content-distribution scheme was proposed, where on-board units (OBUs) were allowed to communicate with other OBUs to obtain missing content. In [30], the collaboration problem of content caching among nodes was studied, and the related problems of utility transfer between nodes under different conditions were also analysed.

To sum up, for a multi-UAV network with caching, the current research mainly concentrates on optimizing one parameter. We try to formulate a network utility jointly considering user satisfaction, transmission energy consumption of UAVs and the cooperative, complementary transmission reliability of UAVs. Then, we further optimize multi-UAV cooperative, complementary transmission to maximize this utility, making the solution closer to a realistic deployment requirement.

## 3. System Model

### 3.1. Network Scenario

**Figure 1:**
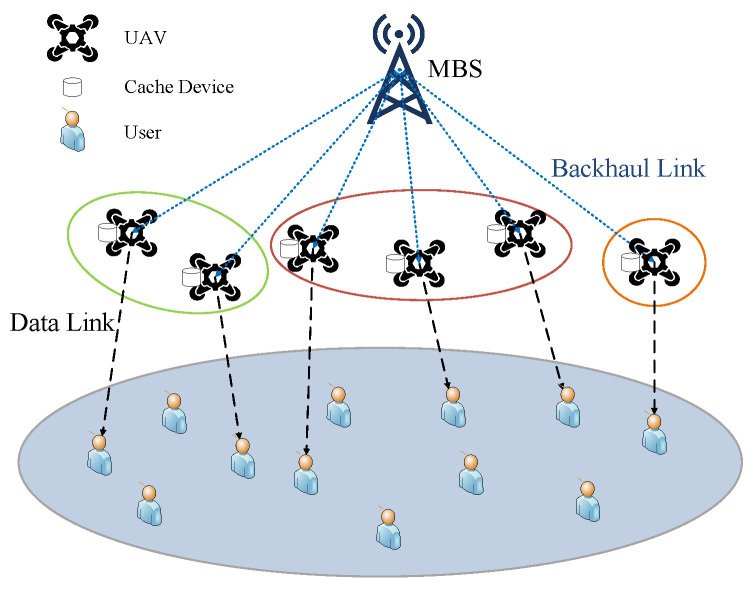
Cache-enabled, UAV-assisted network.

An example of a cache-enabled, UAV-assisted cellular network consisting of an MBS and a set of UAVs is shown in Figure 1. The UAVs overlay on top of a macrocell, forming a hierarchical cell structure. Due to the limited cache capacity and battery life of each UAV, we assume that each UAV can cooperate with nearby UAVs to exchange cached content to better meet the demands of its associated users.

We define the set of one MBS as {0}, the set of UAVs as N={1,2,⋯,N} and the set of users as U={1,2,⋯,U}. The cache capacity of each UAV is uniformly set to be *Q*. UAVs obtain content from the MBS through the backhaul link. The downlink bandwidth of the wireless access network is denoted as *B*, and the bandwidth of the wireless backhaul link is depicted by $B_{b}$. The distribution of users is modeled as a homogeneous Poisson point process (HPPP) with density ψ on a two-dimensional plane. The location of a user *u* is denoted as $w_{u}=\left(x_{u},y_{u}\right)$. The location of the MBS is expressed as $w_{0}=\left(x_{0},y_{0}\right)$. For analysis simplification, we further suppose that all UAVs fly at a given height of *H*. Therefore, the location of a UAV can be depicted as $w_{n}=\left(x_{n},y_{n},H\right)$. The other key variables used in this paper are summarized in Table 1.

### 3.2. Transmission Model

The transmission channel models involve three kinds of links: UAV-to-User, MBS-to-UAV, and UAV-to-UAV. We assume that UAVs and users are static during data transmission.

(1) UAV-to-User link: The air-to-ground pathloss between a low altitude UAV and a user can be predicted by a statistical propagation model [31]. Therefore, the pathloss for Line-of-Sight (*LoS*) and Non-Line-of-Sight (*NLoS*) links, respectively, are expressed as:

(1) $$\begin{array}{c} PL_{n,u}^{LoS}=20\log\left(4\pi fd_{n,u}/c\right)+\eta_{LoS},\;\;PL_{n,u}^{NLoS}=20\log\left(4\pi fd_{n,u}/c\right)+\eta_{NLoS} \end{array}$$

where $d_{n,u}$ denotes the distance between UAV *n* and user *u*, *f* represents the carrier frequency and *c* is the speed of light. Variables $\eta_{LoS}$ and $\eta_{NLoS}$ represent the attenuation factors for *LoS* and *NLoS* links, respectively. Further considering that the *LoS* transmission probability and the *NLoS* transmission probability depend on the density and the height of buildings, the environment, and the elevation angle between UAV and user, they can be respectively expressed as [21]:

(2) $$\begin{array}{c} Pr_{n,u}^{LoS}={\left(1+X\exp\left(-Y\left[\theta_{n,u}-X\right]\right)\right)}^{-1},\;\;Pr_{n,u}^{NLoS}=1-Pr_{n,u}^{LoS} \end{array}$$

where *X* and *Y* are constants depending on environmental factors (urban, suburban, dense urban, etc.); $\theta_{n,u}=\tan^{-1}\left(H/r_{n,u}\right)$ is the elevation angle between UAV *n* and user *u*, where $r_{n,u}=\sqrt{{\left(x_{n}-x_{u}\right)}^{2}+{\left(y_{n}-y_{u}\right)}^{2}}$ is the horizontal distance between UAV *n* and user *u*. Therefore, the average pathloss from UAV *n* to user *u* can be given by [32]:

(3) $$\begin{array}{cc} PL_{n,u} & =\left(Pr_{n,u}^{LoS}\times PL_{n,u}^{LoS}\right)+\left(Pr_{n,u}^{NLoS}\times PL_{n,u}^{NLoS}\right)\\ \; & =\frac{A}{1+X\exp\left(-Y\left[\frac{180}{\pi }\tan^{-1}\left(\frac{H}{r_{n,u}}\right)-X\right]\right)}+10\log\left(H^{2}+{r_{n,u}}^{2}\right)+B \end{array}$$

where $A=\eta_{LoS}-\eta_{NLoS}$ and $B=20\log\left(4\pi fd_{n,u}/c\right)+\eta_{NLoS}$. We assume that the available bandwidth of UAVs is divided equally among users. Based on the transmission model, the signal-to-noise ratio (SNR) is $\gamma_{n,u}=\frac{P_{n}10^{-PL_{n,u}/10}}{\sigma^{2}}$, where $P_{n}$ is the transmit power of UAV *n*, and $\sigma^{2}$ is the variance of the Gaussian noise.

(2) MBS-to-UAV link: The pathlosses of *LoS* link and *NLoS* link from MBS to UAV *n* are given respectively as follows:

(4) $$\begin{array}{c} PL_{0,n}^{LoS}={d_{0,n}}^{-\alpha },\;\;PL_{0,n}^{NLoS}=\zeta {d_{0,n}}^{-\alpha } \end{array}$$

where $d_{0,n}$ denotes the distance between MBS and UAV *n*, α is the pathloss exponent, and ζ is the excessive pathloss coefficient for the *NLoS* link. The *LoS* probability and *NLoS* probability are expressed respectively as below:

(5) $$\begin{array}{c} Pr_{0,n}^{LoS}={\left(1+X\exp\left(-Y\left[\theta_{0,n}-X\right]\right)\right)}^{-1},\;\;Pr_{0,n}^{NLoS}=1-Pr_{0,n}^{LoS} \end{array}$$

where $\theta_{0,n}=\tan^{-1}\left(\frac{H}{r_{0,n}}\right)$, $r_{0,n}$ is the horizontal distance between UAV *n* and MBS. Therefore, the average pathloss from MBS to UAV *n* can be derived as below:

(6) $$\begin{array}{c} PL_{0,n}=\left(Pr_{0,n}^{LoS}\times PL_{0,n}^{LoS}\right)+\left(Pr_{0,n}^{NLoS}\times PL_{0,n}^{NLoS}\right) \end{array}$$

Based on the transmission model, the SNR is $\gamma_{0,n}=\frac{P_{0}10^{-PL_{0,n}/10}}{\sigma^{2}}$, where $P_{0}$ is the transmit power of the MBS.

(3) UAV-to-UAV link: Since the distance between UAVs is only a few hundred meters, the cooperation link between UAVs can adopt a WiFi communication link. Then, the pathloss between UAVs can be calculated by referring to the free space propagation model and be described as below:

(7) $$\begin{array}{c} PL_{n,n^{{}^{\prime }}}=32.44+20\mathrm{lg}f_{w}+20\mathrm{lg}d_{n,n^{{}^{\prime }}} \end{array}$$

where $f_{w}$ is the operating frequency of UAV and $d_{n,n^{{}^{\prime }}}$ is the distance between UAV *n* and UAV $n^{\prime }$. So the SNR is $\gamma_{n,n^{{}^{\prime }}}=\frac{P_{n}10^{-PL_{n,n^{{}^{\prime }}}/10}}{\sigma^{2}}$.

### 3.3. Content Model

In the cache-enabled, UAV-assisted cellular network, we assume that each UAV is equipped with limited cache capacity, which can proactively cache a certain amount of content in its memory. If the requested content of the user is saved in the local cache of its serving UAV, this content can be transmitted to this user via wireless link directly. If the requested content is not be cached at the user’s associated UAV, but is cached at other UAVs, the content can be delivered to the user by cooperative link between UAVs. Otherwise, it should be fetched from the MBS by its serving UAV via wireless backhaul link and then transmitted to the user via radio downlink of its serving UAV. All the content is assumed available at the MBS and constitutes a content library defined as F={1,2,⋯,F}. For analysis simplification, the size of each item of content is set to be equal and denoted as *S*.

The popularity distribution of the content is assumed to remain static over a certain period of time. Content popularity can be characterized by Zipf distribution [33] and denoted as

(8) $$q_{f}=\frac{1/f^{\beta }}{\sum_{i=1}^{F}1/i^{\beta }}$$

where the exponent β>0 characterizes the popularity of content *f*. A larger β implies more frequent requests for the content, which means the more popular content accounts for the majority of content requests. Then the content popularity of all the content can be expressed by the vector $Qu=\left[q_{1},q_{2},\ldots ,q_{f},\ldots ,q_{F}\right]$.

## 4. User-Clustering-Based Caching Strategy

In this section, a caching strategy based on user clustering is proposed to make full use of UAV cache space. First, a user-clustering algorithm based on user similarity is proposed. Then, the caching strategy for each UAV is designed based on user preference and content popularity after the determination of service users of each UAV.

### 4.1. Similarity-Based User Clustering

In this subsection, we consider user clustering, where each user is associated with a specific UAV for better service. Contrary to traditional location-based clustering methods, user similarity is developed in order to group users. The similarity between users is assessed by two factors: content preference similarity and distance similarity. Assuming that $P_{u}$ is the content preference distribution of user *u*, and $P_{v}$ is the content preference distribution of user *v* (v≠u), the content preference similarity between these two users can be calculated according to a cosine similarity metric and expressed as $sim1\left(u,v\right)=\frac{P_{u}\cdot P_{v}}{∥P_{u}∥∥P_{v}∥}$. The distance similarity between two users is evaluated by the physical distance between them and can be defined as $sim2\left(u,v\right)=1-\frac{dist_{u,v}}{max_{u,v\in U}dist_{u,v}}\in \left[0,1\right]$. Taking the above two kinds of similarities into account, the user similarity between user *u* and *v* can be derived as:

(9) $$\begin{array}{c} sim\left(u,v\right)=sim1{\left(u,v\right)}^{\alpha_{1}}\cdot sim2{\left(u,v\right)}^{\alpha_{2}} \end{array}$$

where $\alpha_{1}$ and $\alpha_{2}$ indicate the weighting coefficient of the two metrics. Then, based on user similarity and the total number of UAVs, the user clustering algorithm is described in Algorithm 1 via a spectral clustering (SC) algorithm. After user clustering, it is easier to group additional users with similar content preferences and close proximity. Then, UAVs can be deployed at the center of each user cluster on a two-dimensional plane.
**Algorithm 1** Similarity-Based User Clustering Algorithm1:Initialization: the number of UAV *N*; the similarity matrix Sim defined as in (9).2:Build the adjacency matrix W=Sim, and calculate the degree matrix D with diagonal element $d_{i}=\sum_{j=1}^{U}{sim}_{i,j}$.3:Calculate Laplace matrix L=D−W.4:Calculate normalized Laplace graph matrix $L_{norm}=D^{-1/2}LD^{-1/2}$.5:Pick a number of *N* eigenvalues of $\lambda_{1}\leq \lambda_{2}\leq \ldots \leq \lambda_{N}$ of $L_{norm}$.6:Calculate the *N* smallest eigenvectors $z_{1}\leq z_{2}\leq \ldots \leq z_{N}$.7:Let the Z matrix have the eigenvectors $z_{1},z_{2},\ldots ,z_{N}$ as columns.8:Use K-means clustering to cluster the rows of the matrix Z.9:Output: the division result of cluster $C\left(c_{1},c_{2},\ldots c_{N}\right)$.

### 4.2. User-Clustering-Based Caching Strategy for UAVs

After user clustering and UAV placement, the caching strategy of each UAV can be further optimized to meet its users’ content requirements as much as possible. The content caching strategy is denoted by matrix *X* of size N×F, where $x_{n,f}=1$ represents that content *f* is cached at UAV *n*, otherwise $x_{n,f}=0$.

Considering that a user’s request for content is not only affected by the the content’s popularity, but also by the interests of the user, in our proposed UAV caching strategy, content popularity and the interests of different users are jointly taken into account for content caching at UAVs. For further clarification, content popularity is measured by the statistical results of the interests of all users, which describes the average level of interests of all users across the network. The distribution of content popularity can be expressed by the Zipf model [33], as depicted in formula (8). Further, the content interests of a user is for a single user and is related to the user’s background (such as age, gender, occupation, etc.) The content interests of users within a UAV group is a different concept that content popularity measured across the network. We assume that each user’s interest in content is independently and identically distributed. The interests of user *u* in content *f* is characterized by a probability value $p_{u,f}$, where $0<p_{u,f}<1$. Then, the content preference vector of user *u* can be denoted by $P_{u}=\left[p_{u,1},p_{u,2},\cdots ,p_{u,F}\right]$. A binary indicator $r_{u,f}\in \left\{0,1\right\}$ is further defined to represent whether user *u* requests content *f* or not, where $r_{u,f}=1$ when user *u* requests content *f*, otherwise $r_{u,f}=0$.

Thus, taking the above two influencing factors into account, the caching probability of content *f* for UAV *n* is given as:

(10) $$c_{n,f}=\sum_{u\in U^{n}}\left(\gamma_{1}\cdot q_{u,f}+\gamma_{2}\cdot p_{u,f}\right)$$

where $\gamma_{1}$ and $\gamma_{2}$ are the weighting coefficients for content popularity and the content interests of the user, respectively; $q_{u,f}=q_{f}$ represents the content popularity is equal for each user in a period of time; $U^{n}$ means the set of users associated with UAV *n*. Assuming that each UAV can at most cache *Q* content, after sorting the caching probabilities of all content in descending order for each UAV, the top *Q* content can be selected for caching in each UAV.

## 5. Multi-UAV Cooperative, Complementary Transmission Based on Coalition Formation Game

Due to the fact that the caching capacity of one UAV is limited, it is necessary to study multi-UAV cooperative content transmission. In this section, we further investigate multi-UAV coalition formation to realize multi-UAV cooperative content transmission and system utility maximization based on CFG. Then, when a user is unable to find requested content in his/her associated UAV, but the content is cached in other UAVs in the same coalition, the user-associated UAV can then obtain the requested content from other UAVs and deliver it to the user.

### 5.1. System Utility

System utility is formulated by considering user satisfaction, transmission energy consumption of UAVs, and the reliability of cooperative, complementary UAV transmission.

#### 5.1.1. User Satisfaction

To characterize user satisfaction, a mean opinion score (MOS) model is utilized as a measure of quality of service (QoS) for the network services, which takes transmission delay into account and is denoted as below:

(11) $$\begin{array}{c} MOS_{n,u,f}=C_{1}\ln\left(\frac{1}{D_{n,u,f}}\right)+C_{2} \end{array}$$

where $D_{n,u,f}$ represents content transmission delay, $C_{1}$ and $C_{2}$ are both constants, and $C_{1}>0$. It is clear that the smaller the transmission delay, the larger the MOS. For the transmission delay, three kinds of links are considered: UAV-to-User, MBS-to-UAV and UAV-to-UAV. The bandwidth of each kind of link is evenly divided for analysis simplification.

Therefore, for the UAV-to-User link, the downlink data rate and one item of content with size *S*, the transmission delay from the UAV *n* to the user *u* is given as follows:

(12) $$\begin{array}{c} R_{n,u}=\frac{B/N}{|U^{n}|}\log_{2}\left(1+\gamma_{n,u}\right) \end{array}$$

(13) $$\begin{array}{c} D_{n,u}=S/R_{n,u} \end{array}$$

For the MBS-to-UAV link, for the backhaul data rate and one item of content with size *S*, the transmission delay from the MBS 0 to the UAV *n* is given as:

(14) $$\begin{array}{c} R_{0,n}=\frac{B_{b}}{N}\log_{2}\left(1+\gamma_{0,n}\right) \end{array}$$

(15) $$\begin{array}{c} D_{0,n}=S/R_{0,n} \end{array}$$

where $B_{b}$ is the backhaul bandwidth between UAVs and the MBS.

As for the UAV-to-UAV link, we define $B_{c}$ as the bandwidth of the cooperation link, so for the cooperation link data rate and one item of content with size *S*, the transmission delay between UAVs *n* and $n^{{}^{\prime }}$ is estimated as:

(16) $$\begin{array}{c} R_{n,n^{\prime }}=\frac{B_{c}}{N}\log_{2}\left(1+\gamma_{n,n^{\prime }}\right) \end{array}$$

(17) $$\begin{array}{c} D_{n,n^{{}^{\prime }}}=S/R_{n,n^{{}^{\prime }}} \end{array}$$

Therefore, the transmission delay for user *u* of UAV *n* requesting content *f* can be expressed as:

(18) $$\begin{array}{c} D_{n,u,f}=D_{n,u}+\left(1-x_{n,f}\right)\left[x_{n^{\prime },f}D_{n,n^{\prime }}+\left(1-x_{n^{\prime },f}\right)D_{0,n}\right],\;\forall n,n^{\prime }\in N,n\neq n^{\prime } \end{array}$$

where $x_{n,f}\in \left[0,1\right]$ and $x_{n^{{}^{\prime }},f}\in \left[0,1\right]$ represent the cache binary indicators of UAV *n* and UAV $n^{\prime }$, respectively. Referring to Formula (11), all the users’ satisfaction associated with UAV *n* can be derived as:

(19) $$\begin{array}{c} MOS_{n}=\sum_{u\in U^{n}}\sum_{f\in F}r_{n,u,f}MOS_{n,u,f} \end{array}$$

where $r_{n,u,f}\in \left[0,1\right]$ is the binary indicator of user *u* of UAV *n* requesting content *f*.

#### 5.1.2. Transmission Energy Consumption

In this subsection, we discuss network energy consumption for content transmission. For the UAV-to-User link, MBS-to-UAV link and UAV-to-UAV link, respectively, the transmission energy of one item of content with size *S* is given as follows:

(20) $$\begin{array}{c} E_{n,u}=P_{n}\frac{S}{R_{n,u}},\;\;E_{0,n}=P_{0}\frac{S}{R_{0,n}},\;\;E_{n,n^{{}^{\prime }}}=P_{n}\frac{S}{R_{n,n^{{}^{\prime }}}} \end{array}$$

Therefore, for all users of UAV *n*, the total transmission energy consumption of UAV *n* in the process of delivering content to users can be derived as:

(21) $$\begin{array}{c} E_{n}=\sum_{u\in U^{n}}\sum_{f\in F}r_{n,u,f}\left[E_{n,u}+\left(1-x_{n,f}\right)\left(x_{n^{{}^{\prime }},f}E_{n,n^{{}^{\prime }}}+\left(1-x_{n^{{}^{\prime }},f}\right)E_{0,n}\right)\right] \end{array}$$

#### 5.1.3. Cooperative, Complementary Transmission Reliability of UAVs

In the majority of existing techniques, it is assumed that the UAVs are perfectly operated during the transmission task lifetime, this is obviously not a realistic assumption, as UAV operation can be interrupted due to exhaustion of the battery. Thus, we define the transmission reliability of a UAV as the successful transmission of content to its users without the link connection interrupted.

Due to the fact that content requests of different users are independent of each other, the transmission process between a UAV and each of its user is also independent. Considering that the transmission process depends on the length of the transmission time period, that is, the transmission delay, content transmission is a Poisson process. Thus, transmission reliability can be described based on the Poisson model. Further, considering the probability of transmission failure, the transmission reliability of UAV *n* transmitting content to the users it is serving can be expressed as:

(22) $$\begin{array}{c} R_{n}=\exp\left(-\sum_{u\in U^{n}}\sum_{f\in F}r_{n,u,f}\lambda_{n,u,f}D_{n,u,f}\right) \end{array}$$

where $D_{n,u,f}$ is content transmission delay, which is also the execution time of UAV *n* serving user *n*, and $\lambda_{n,u,f}$ is the failure rate of UAV *n* delivering content *f* to user *u*. Thus, cooperative, complementary transmission reliability of coalition $S_{k}$ is calculated as follows:

(23) $$\begin{array}{c} R\left(S_{k}\right)=\prod_{n\in S_{k}}R_{n} \end{array}$$

#### 5.1.4. System Utility and Problem Formulation

We regard user satisfaction and system cooperative, complementary transmission reliability as the benefits of the network, and take transmission energy consumption as the cost of the network. Then, we define the utility of UAV *n* as:

(24) $$\begin{array}{c} u_{n}=\frac{MOS_{n}+R_{n}}{E_{n}} \end{array}$$

which represents the benefits obtained per unit of energy consumed. For a multi-UAV network, UAVs can form multiple coalitions to realize cooperative, complementary transmission. Thus, for any one coalition $S_{k}$, its utility can be given as:

(25) $$\begin{array}{cc} U\left(S_{k}\right) & =\frac{MOS\left(S_{k}\right)+R\left(S_{k}\right)}{E\left(S_{k}\right)}=\frac{\sum_{n\in S_{k}}MOS_{n}+\prod_{n\in S_{k}}R_{n}}{\sum_{n\in S_{k}}E_{n}} \end{array}$$

where $\sum_{n\in S_{k}}MOS_{n}$ means total user satisfaction in this coalition, $\prod_{n\in S_{k}}R_{n}$ is the cooperative, complementary transmission reliability in this coalition, and $\sum_{n\in S_{k}}E_{n}$ is the total transmission energy consumption of this coalition.

Therefore, the system utility of the whole network can be depicted as:

(26) $$U_{sys}=\sum_{S_{k}\in \Pi }U\left(S_{k}\right)$$

where Π represents the coalition set that contains all coalitions. Our goal is to maximize the system utility of the whole network. Hence, the optimization problem can be formulated as below:

(27) $$\begin{array}{cc} maximize & \;\;U_{sys}=\sum_{S_{k}\in \Pi }U\left(S_{k}\right)\\ s.t. & \;\;C1:x_{n,f}\in \left\{0,1\right\},r_{n,u,f}\in \left\{0,1\right\}\\ \; & \;\;C2:\sum_{f=1}^{F}x_{n,f}\leq Q_{n} \end{array}$$

where C1 implies that the content cache variable $x_{n,f}$ and user request variable $r_{n,u,f}$ are binary indicator variables; C2 restricts the number of items cached to not exceed the cache space of UAV *n*.

### 5.2. UAV Coalition Optimization for System Utility Maximization

In this section, we model the UAVs cooperative, complementary transmission problem as a coalition formation game (CFG), which provides an outstanding tool to reveal the coalition formation process.

#### 5.2.1. Coalition Formation Game

Cooperative content delivery among UAVs can be modeled as G = $\left\{a_{n},A,\Pi ,u_{n},\right.\left.U\left(S_{k}\right),U_{sys}\right\}$, where $a_{n}$ is the decision of UAV *n*, *A* is the set of all UAV decisions, and $u_{n}$ is the utility function of UAV *n*. Then, we give some definitions that are applied in CFGs.

**Definition** **1** (Coalition structure)**.** *The set $\Pi =\left\{S_{1},S_{2},\cdots ,S_{k},\cdots ,S_{K}\right\}$ is called a coalition structure of the UAV set N, where $S_{k}\cap S_{k^{{}^{\prime }}}=⌀$, $k\neq k^{{}^{\prime }}$ and $\cup_{k=1}^{K}S_{k}=N$.*

In particular, if $\left\{n\right\}$ (n∈N) is a special coalition that contains only one UAV, then it cannot participate in cooperative transmission with other UAVs. On the contrary, if a coalition includes all UAV players, then it is called the grand coalition. When any coalition $S_{k}$ in the coalition structure Π no longer changes, the coalition structure Π is stable.

**Definition** **2** (Preference order)**.** *For any UAV n∈N, ${≻}_{n}$ is defined as a complete, reflexive and transitive binary relation over the set of all feasible and preferred coalitions that the UAV can possibly join.*

For UAV *n*, it will join or leave a coalition based on the preference order. That is to say, given two coalitions $S_{1}\subseteq \Pi$ and $S_{2}\subseteq \Pi$, if $S_{1}{≻}_{n}S_{2}$, UAV *n* is more willing to join coalition $S_{1}$ than coalition $S_{2}$. The preference order can influence CFG convergence and the final coalition structure. Two traditional preference orders that are commonly used in CFG are described as follows.

**Definition** **3** (Pareto order)**.** *For any UAV n, ∀n∈N, and any two coalitions $S_{i}\subseteq \Pi ,S_{j}\subseteq \Pi$, we say that*

(28) $$\begin{array}{cc} S_{i}{≻}_{n}S_{j}\Leftrightarrow & u_{n}\left(S_{i}\right)>u_{n}\left(S_{j}\right)\wedge u_{k}\left(S_{i}\right)\geq u_{k}\left(S_{i}\setminus \left\{n\right\}\right),\forall k\in S_{i}\setminus \left\{n\right\}\\ \; & \wedge u_{k}\left(S_{j}\right)\leq u_{k}\left(S_{j}\setminus \left\{n\right\}\right),\;\forall k\in S_{j}\setminus \left\{n\right\} \end{array}$$

*where $u_{n}\left(S_{i}\right)$ and $u_{n}\left(S_{j}\right)$ are the utilities of UAV n joining coalition $S_{i}$ and $S_{j}$, respectively. The Pareto order defined in (28) ensures that UAV n will not damage other UAVs’ utilities when increasing its own utility by joining or leaving a coalition [29].*

**Definition** **4** (Selfish order)**.** *For any UAV n, ∀n∈N, and any two coalitions $S_{i}\subseteq \Pi ,S_{j}\subseteq \Pi$, we say that*

(29) $$\begin{array}{c} S_{i}{≻}_{n}S_{j}\Leftrightarrow u_{n}\left(S_{i}\right)>u_{n}\left(S_{j}\right) \end{array}$$

In Selfish order, a UAV will pursue its own higher utility, regardless of the utilities of other UAV players, which may deteriorate the utilities of other UAVs and even degrade the entire system. Pareto order, on the other hand, considers all UAV players’ utilities in both the current coalition structure and the new coalition structure. This results in UAVs rarely leaving their current coalition due to the strong restriction once they joins a coalition. Thus, system performance will fall into partial optimization.

To overcome the shortcomings of the above two preference orders, we propose a Cooperation-order-based coalition formation game (CO-CFG) to maximize the whole network utility.

**Definition** **5** (Cooperation order)**.** *For any UAV n, ∀n∈N, $S_{j}\subseteq \Pi$ is its original coalition, and $S_{i}\subseteq \Pi$ is a new coalition it intends to join, we say that*

(30) $$\begin{array}{c} S_{i}{≻}_{n}S_{j}\Leftrightarrow \sum_{n\in S_{i}}u_{n}\left(S_{i}\right)+\sum_{k\in S_{j}\setminus \left\{n\right\}}u_{k}\left(S_{j}\setminus \left\{n\right\}\right)>\sum_{n\in S_{j}}u_{n}\left(S_{j}\right)+\sum_{k\in S_{i}\setminus \left\{n\right\}}u_{k}\left(S_{i}\setminus \left\{n\right\}\right) \end{array}$$

*where $\sum_{n\in S_{i}}u_{n}\left(S_{i}\right)+\sum_{k\in S_{j}\setminus \left\{n\right\}}u_{k}\left(S_{j}\setminus \left\{n\right\}\right)$ is the sum of the utility of the new coalition and the utility of the original coalition after UAV n joins coalition $S_{i}$, and $\sum_{n\in S_{j}}u_{n}\left(S_{j}\right)+\sum_{k\in S_{i}\setminus \left\{n\right\}}u_{k}\left(S_{i}\setminus \left\{n\right\}\right)$ is the sum of the utility of the new coalition and the utility of the original coalition before UAV n joins coalition $S_{i}$. According to the Cooperation order, a UAV will switch coalitions if it results in an overall increase in utility.*

Based on preference order, the coalition exchange operation for the coalition formation game is provided as follows.

**Definition** **6** (Exchange operation)**.** Given a coalition structure $\Pi =\left\{S_{1},S_{2},\cdots ,S_{k},\cdots ,S_{K}\right\}$, if the system utility of the UAV joining the new coalition is greater than that of the UAV staying in the original coalition, the exchange operation will occur; that is, UAV player $n\in S_{j}$ will leave the current coalition $S_{j}$ and join a new coalition $S_{i}$. Thus, the current coalition structure *Π* will be transformed into a new structure $\Pi^{*}=\left(\Pi \setminus \left\{S_{i},S_{j}\right\}\right)\cup \left\{S_{j}\setminus \left\{n\right\},S_{i}\cup \left\{n\right\}\right\}$.

The exchange operation provides a mechanism by which a UAV can leave its original coalition and join a new coalition when it meets the exchange conditions. UAVs in the coalition can exchange information constantly through the above-mentioned three preference orders.

Then, we prove the stability of the coalition structure under the preference orders.

**Definition** **7** (Stable coalition structure [28])**.** *A coalition structure *Π* is stable if no player can improve its utility through changing its coalition unilaterally with the corresponding order. That is,*

(31) $$\begin{array}{c} u_{n}\left(a_{n},a_{-n}\right)\geq u_{n}\left(a_{n}^{\prime },a_{-n}\right),\;\forall n\in N,a_{n}\neq a_{n}^{\prime } \end{array}$$

*where $a_{n}$ and $a_{n}^{\prime }$ mean the actions of UAV n, and $a_{-n}$ represents the actions of other UAVs except UAV n.*

**Theorem** **1.** *The CFG based on Pareto order and Selfish order can converge to a stable coalition structure.*

**Theorem** **2.** *The CFG based on Cooperation order has at least one stable coalition structure.*

See Appendix A for proofs of Theorems 1 and 2.

#### 5.2.2. CO-CFG Algorithm

In the above analysis, the definition of the cooperation rule is given; further, coalition structure stability is proven. Then, we further propose the Cooperation-order-based Coalition-Formation-Game (CO-CFG) algorithm. In the coalition game process, in order to prevent UAVs from repeatedly joining the same coalition and to ensure faster convergence, we first define an historical selection set $H_{n}$ and a candidate set ${Ca}_{n}$ for UAV *n*. The historical selection set $H_{n}$ contains all the coalitions that had ever formed. Therefore, for the Cooperation order, not only must the conditions be met, but the transition to a new coalition can only be carried out if the proposed configuration is not in the historical selection set. At the same time, the new coalition is added to the candidate set ${Ca}_{n}$. In the candidate set, the optimal coalition that maximizes the system utility of the whole network is selected as the coalition that UAV *n* finally joins, and then, the optimal coalition is added to the historical selection set $H_{n}$. Details of the proposed CO-CFG algorithm are described as Algorithm 2.
**Algorithm 2** CO-CFG Algorithm1:Initialize: The set of UAVs is $\left\{1,2,3,\cdots ,N\right\}$, initial coalition structure is $\Pi^{0}=\left\{\left\{1\right\},\left\{2\right\},\cdots ,\left\{N\right\}\right\}$ and the historical selection set is ${\left\{H_{n}\right\}}_{n\in N}=⌀$.2:**repeat**3:   Step 1: Calculate the utility $u_{n}$ of UAV *n*, the utility $U_{S_{k}}$ of coalition and the total system utility under the current coalition structure.4:   Step 2: UAV *n* selects a coalition from the existing coalitions, and calculates its own utility, coalition utility and system utility after joining. Judging whether UAV *n* meets the following transfer conditions after it transfers from the current coalition $S_{j}$ to the new coalition $S_{i}$:5:      (a) The utility of UAV *n* is increasing, i.e., $u_{n}\left(S_{i}\right)>u_{n}\left(S_{j}\right)$;6:      (b) The system utility is increasing as defined in cooperation order;7:      (c) The new coalition $S_{i}$ does not exist in the historical selection set.8:       If the transfer conditions are met, the coalition $S_{i}$ is added to the candidate set ${Ca}_{n}$. Otherwise, a new coalition is selected to join.9:   Step 3: If $Ca_{n}\neq ⌀$, UAV *n* joins the optimal coalition $S_{opt}$ which maximizes system utility, and $S_{opt}$ is added to $H_{n}$.10:   Step 4: Update coalition structure Π. Otherwise, the historical selection set will not be changed.11:**until** the historical selection set no longer changes.

## 6. Simulation Results

In this section, numerical simulation experiments are carried out to evaluate the performance of our proposed algorithms. We consider a network coverage area within a square region of 500×500 m${}^{2}$. The distribution of users follows the HPPP model. Unless specified otherwise, the network parameters are summarized in Table 2.

**Figure 2:**
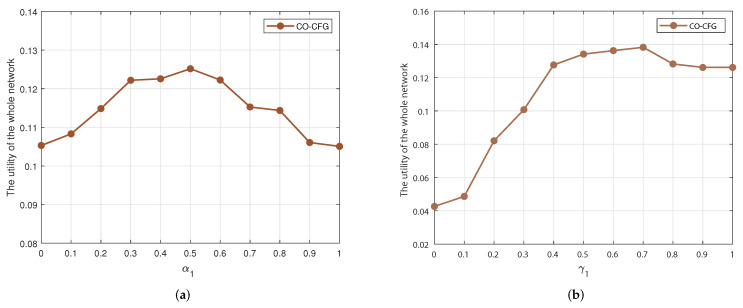
Impact of different (**a**) $\alpha_{1}$ and (**b**) $\gamma_{1}$ on the utility of the whole network. (**a**) The effect of $\alpha_{1}$ on the utility of the whole network. (**b**) The effect of $\gamma_{1}$ on the utility of the whole network.

First of all, the weighting coefficients $\alpha_{1}$ and $\alpha_{2}$ in the similarity-based user clustering algorithm and the weighting coefficients $\gamma_{1}$ and $\gamma_{2}$ in the user-clustering-based caching strategy are determined by simulation analysis, as shown in Figure 2. In similarity-based user clustering, parameter $\alpha_{1}$ and $\alpha_{2}$ indicate the weighting coefficients of content-preference similarity and distance similarity, respectively. The utility of the whole network versus $\alpha_{1}$ is shown in Figure 2a, where $\alpha_{2}=1-\alpha_{1}$. It can be seen that performance is best when content preference similarity and distance similarity are considered evenly, i.e., $\alpha_{1}=\alpha_{2}=0.5$. Further, for the user-clustering-based caching strategy, $\gamma_{1}$ and $\gamma_{2}$ refer to the weighting coefficients for content popularity and content interests of the user, respectively, where $\gamma_{2}=1-\gamma_{1}$. From Figure 2b, we can see that when $\gamma_{1}=0.7$ and $\gamma_{2}=0.3$, the utility of the whole network is maximized.

### 6.1. Performance of User-Clustering-Based Caching Strategy

**Figure 3:**
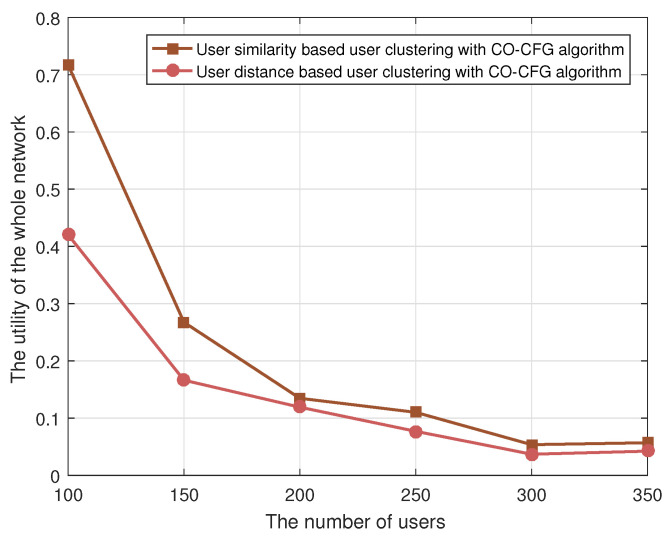
The utility of the whole network with varying the user clustering algorithm.

As shown in Figure 3, the performance of Algorithm 1, i.e., our proposed similarity-based user clustering algorithm, is compared with the distance-based user clustering algorithm, where the CO-CFG algorithm is adopted for UAVs’ cooperative, complementary transmission and to calculate the utility of the whole network. We can see that the performance of our proposed similarity-based user clustering is better than that of the distance-based user clustering. This is because the similarity-based user clustering algorithm jointly considers user preferences and distance, which makes better use of the UAV’s cache space for content caching, achieving better results. On the other hand, the distance-based user clustering algorithm generally clusters users based on the distance between them, which makes it difficult to explore the content preference relationship among users, and thus reduces the content request hit probability of the cached resources.

**Figure 4:**
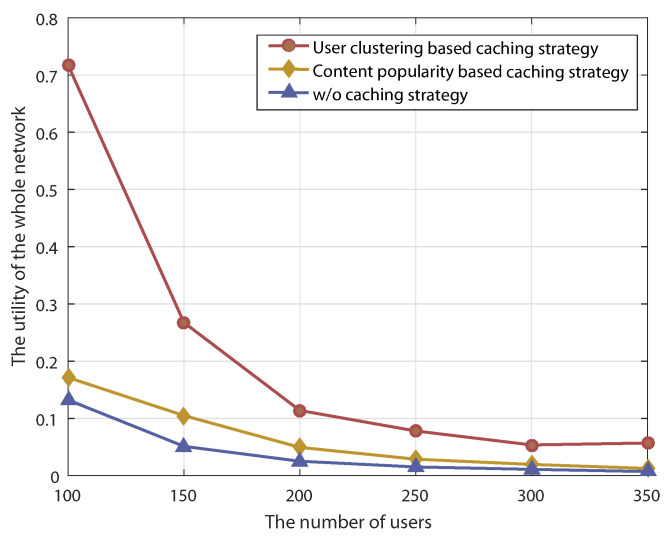
The utility of the whole network for different UAV caching strategies.

As shown in Figure 4, the performance of our proposed user-clustering-based caching strategy is compared with that of the content-popularity-based caching strategy and a strategy without caching. The results show that the performance of these two caching strategy is better than that of the strategy without caching. This is because that cache-enabled UAV can directly transmit the required content to users, which effectively reduces access delay and energy consumption, improving whole-network utility. Furthermore, the performance of our proposed user-clustering-based caching strategy is better than that of the content-popularity-based caching strategy. This is due to the fact that our proposed caching strategy is optimized based on clustering users with similar interests, which allows the content cached in UAVs to more effectively meet the preferences of users. As the number of users increases, the network utility of all three strategies declines accordingly because the increase of users leads to more serious congestion of the UAV-to-User link, increasing transmission delay and energy consumption due to the limitation of bandwidth resources.

### 6.2. Performance of Multi-UAV Cooperative, Complementary Transmission

In this subsection, we verify the performance of our proposed CO-CFG algorithm for multi-UAV cooperative, complementary transmission in terms of number of UAVs, number of users and UAV cache space. The algorithms compared include the Pareto-order-based coalition-formation-game (PO-CFG) algorithm, Selfish-order-based coalition-formation-game (SO-CFG) algorithm and the non-cooperation scheme.

**Figure 5:**
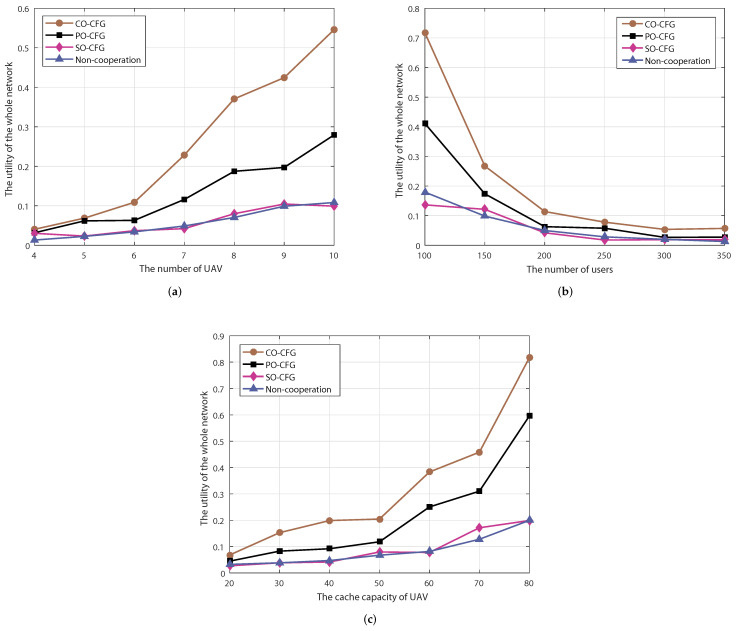
The utility of the whole network with (**a**) differing numbers of UAVs, (**b**) differing numbers of users and (**c**) varying UAV cache capacity.

The utility of the whole network versus the number of UAVs is depicted in Figure 5a, where the number of users is equal to 200 and the number of cached items is 40. It is apparent that the network utility achieved by our proposed CO-CFG algorithm is higher than those of the PO-CFG algorithm and the SO-CFG algorithm. The performance of the non-cooperation scheme is the lowest since there is no cooperation between UAVs, and the content requested by users is not cached in the serving UAV and can only be retrieved from the macro base station through the UAV, which increases access delay and energy consumption. By increasing the number of UAVs, the utility of the whole network increases. This is because, with a fixed number of users, increasing the number of UAVs leads to a reduction in the number of users served by a single UAV, making the combination of user clustering, content caching and cooperative, complementary transmission more effective.

The relationship between the utility of the whole network and varying the number of users is described in Figure 5b, where the number of UAVs is 7, and there are 40 items cached. As shown in the figure, our proposed CO-CFG algorithm performs the best out of the four algorithms tested. As the number of users increases, system performance will deteriorate accordingly. This is due to the fact that the increase in the number of users leads to an increase in the UAV load and a related increase in transmission delay and energy consumption, thus resulting in a decline of the whole network utility.

The utility of the whole network for various cache capacities is represented in Figure 5c, where the cache capacity of each UAV ranges from 20 to 80 items, the number of UAVs is set to 7, and the number of users is 200. The simulation results show that the proposed CO-CFG algorithm achieves the highest system utility out of all four algorithms. This is due to the fact that our proposed algorithm with the user-clustering-based caching strategy can take full advantage of each UAV’s cache space. As the cache capacity increases, the utility of the whole network of the proposed CO-CFG, PO-CFG, SO-CFG and non-cooperation scheme improve accordingly because the UAVs can cache more content to better meet the requirements of users.

**Figure 6:**
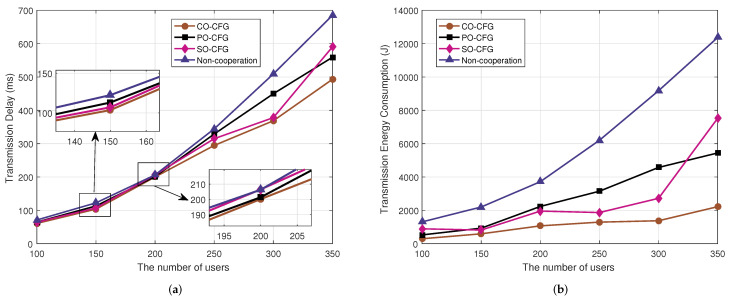
Impact of increasing the number of users on (**a**) system transmission delay and (**b**) system transmission energy consumption.

System transmission delay and energy consumption for various numbers of users are represented in Figure 6a,b, respectively, where the number of UAVs is 7 and the quantity of cached items is 40. We can see that as the number of users increases, the communication link load increases, resulting in increased transmission delay and energy consumption. However, system transmission delay and transmission energy consumption of the CO-CFG algorithm are both lower than those of the PO-CFG, SO-CFG and non-cooperation algorithms.

### 6.3. Convergence Performance

**Figure 7:**
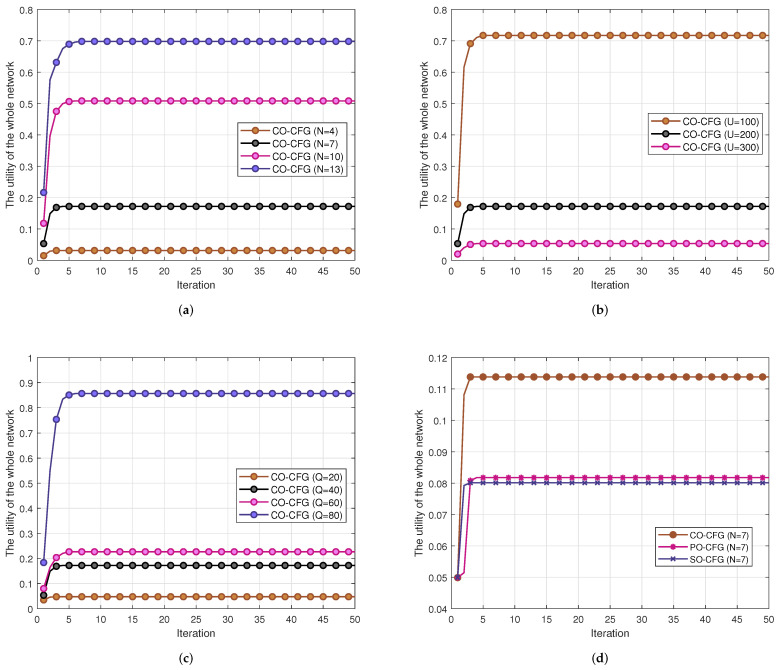
The convergence performance of the proposed CO-CFG algorithm. (**a**) The convergence of the CO-CFG algorithm under different numbers of UAVs. (**b**) The convergence of the CO-CFG algorithm under different numbers of users. (**c**) The convergence of the CO-CFG algorithm under various UAV cache capacities. (**d**) The convergence of the CO-CFG algorithm, PO-CFG algorithm and SO-CFG algorithm.

The convergence of our proposed CO-CFG algorithm with different numbers of UAVs *N*, different number of users *U* and varying UAV cache capacity *Q* is depicted in Figure 7a–c, respectively. We can see that, since we adopt the history set in the algorithm, convergence to the stable state occurs quickly. The comparison of the CO-CFG algorithm with the PO-CFG algorithm and SO-CFG algorithm is described in Figure 7d. It can be seen that all of these three algorithms can converge to a stable state quickly due to the incorporation of the history set in these algorithms, and the performance improvement brought to the CO-CFG algorithm is larger than those of the other two algorithms. On the one hand, since the exchange operation may decrease the utility of other players, a UAV under PO-CFG is often trapped in the original coalition by the other UAV players, which is not good for the utility of the whole network. On the other hand, the SO-CFG only makes a UAV pursue a better utility for itself, which may deteriorate the total utility of the whole network. Our proposed CO-CFG takes both the utility of a single UAV and the whole network into account, resulting in better performance.

## 7. Conclusions

In this paper, we investigate the cooperative, complementary content transmission problem for a multi-UAV network by jointly considering user clustering and cache management. Firstly, similarity-based user clustering is developed, where users’ content preference similarities and distance similarities are both considered. Secondly, the user-clustering-based caching strategy is further studied to better serve users and improve the utilization of caching resources in UAVs, where content popularity and the content preference of users are both taken into account. Finally, we model the multi-UAV cooperative, complementary transmission problem as a coalition-formation game and propose a Cooperation-order-based coalition-formation-game (CO-CFG) algorithm to maximize the whole network utility, which is defined by jointly considering user satisfaction, transmission energy consumption of UAVs and the cooperative, complementary transmission reliability of UAVs. Numerical experiments show that the performance of our proposed similarity-based user-clustering algorithm is better than that of a distance-based user-clustering algorithm; user-clustering-based caching is superior to content-popularity-based caching; and the CO-CFG algorithm performs better than PO-CFG, SO-CFG and Non-cooperation algorithms.

## Figures and Tables

**Table 1:** Key Variables Used in This Paper.

Symbol	Description
*N*	Number of UAVs
*U*	Number of users
*F*	Quantity of content items
*Q*	Cache capacity of UAV
*S*	Size of each content item
*B*	Bandwidth of downlink data link
$B_{b}$	Bandwidth of backhaul link
$B_{c}$	Bandwidth of cooperation link
$x_{n,f}$	Indicator of whether UAV *n* caches content *f*
$r_{u,f}$	Indicator of whether user *u* requests content *f*
$P_{0}$	Transmission power of MBS
$P_{n}$	Transmission power of UAV
*H*	Flight height of UAV
$w_{0},\;w_{n},\;w_{u}$	Two-dimensional plane position of MBS, UAV, user
$d_{0,n},\;d_{n,u},\;d_{n,n^{\prime }}$	Distance between MBS and UAV, UAV and user, UAV and UAV
$PL_{0,n},\;PL_{n,u},\;PL_{n,n^{\prime }}$	pathloss of MBS-to-UAV link, UAV-to-User link, UAV-to-UAV link
$\gamma_{0,n},\;\gamma_{n,u},\;\gamma_{n,n^{\prime }}$	SNR of MBS-to-UAV link, UAV-to-User link, UAV-to-UAV link
$R_{0,n},\;R_{n,u},\;R_{n,n^{\prime }}$	Data rate of MBS-to-UAV link, UAV-to-User link, UAV-to-UAV link
$D_{0,n},\;D_{n,u},\;D_{n,n^{\prime }}$	Delay of MBS-to-UAV link, UAV-to-User link, UAV-to-UAV link

**Table 2:** Simulation Parameters.

Parameter	Value
Quantity of content items	100
UAV height *H*	100 m
UAV transmission power $P_{n}$	30 dBm
MBS transmission power $P_{0}$	43 dBm
Variance of the Gaussian noise $\sigma^{2}$	−174 dBm/Hz
Bandwidth of data link *B*	20 MHz
Bandwidth of backhaul link $B_{b}$	10 MHz
Bandwidth of cooperative link $B_{c}$	10 MHz
Size of content *S*	10 Mbits
Zipf parameter β	0.8
Reliability parameter λ	$5\times 10^{-5}$
Attenuation factors for *LoS* link $\eta_{LoS}$	1.6 dBm
Attenuation factors for *NLoS* link $\eta_{NLoS}$	23 dBm
Carrier frequency *f*	5 GHz
*X*	11.9
*Y*	0.13
pathloss exponent α	2
Excessive pathloss coefficient ζ	100

## Data Availability

Not applicable.

## Abbreviations

The following abbreviations are used in this manuscript:

4G	4th Generation Mobile Networks
5G	5th Generation Mobile Networks
BS	Base Station
MBS	Macro Base Station
SBS	Small Base Station
UAV	Unmanned Aerial Vehicle
EE	Energy Efficiency
D2D	Device-to-Device
HetNets	Heterogeneous Networks
QoE	Quality of Experience
QoS	Quality of Service
IoT	Internet of Thing
HPPP	Homogeneous Poisson Point Process
LoS	Line-of-Sight
NLoS	Non-Line-of-Sight
SNR	Signal-to-Noise Ratio
SC	Spectral Clustering
MOS	Mean Opinion Score
CFG	Coalition Formation Game
CO-CFG	Cooperation-Order-based Coalition Formation Game
PO-CFG	Pareto-Order-based Coalition Formation Game
SO-CFG	Selfish-Order-based Coalition Formation Game
NE	Nash Equilibrium
EPG	Exact Potential Game

## Appendix A

### Appendix A.1

**Proof of Theorem 1.** Assuming$\Pi^{0}$ is the initial coalition structure, when the exchange operation occurs according to Definition 6, there exists a sequence of exchange operations. In the above-mentioned CFG, given a certain number of UAVs, the total number of possible coalitions is finite. According to the Pareto order, the initial coalition structure $\Pi^{0}$ will shift among finite states. If the final state $\Pi^{*}$ is not stable, the exchange operation will always occur. Thus, it will eventually converge to a stable structure $\Pi^{*}$ after finite iterations. Further, more details can be found in [34]. The work proposed the concept of stable partition, and proved that the reflexive comparison relation, that is, the preference order, had stable partition. Therefore, as one of the reflexive comparison relations, Pareto order will converge to a stable structure. As for Selfish order, a UAV may exchange its coalition to pursue higher utility. If $\Pi^{*}$ is not the final stable partition, it contradicts the previous argument. Consequently, the final state must be stable. Therefore, Theorem 1 is proved. □

### Appendix A.2

**Proof of Theorem 2.** We prove the existence of the stable coalition for Cooperation order by introducing Nash Equilibrium (NE) and Exact Potential Game (EPG). □

**Definition** **A1** (Nash Equilibrium [35])**.** *A coalition exchange action $a^{*}=\left(a_{1}^{*},a_{2}^{*},\cdots ,a_{N}^{*}\right)$ is a pure-strategy NE if no UAV player can improve its own utility by changing its coalition with the other UAVs remaining in the same coalition, i.e.,*

(A1) $$\begin{array}{c} u_{n}\left(a_{n}^{*},a_{-n}^{*}\right)\geq u_{n}\left(a_{n},a_{-n}^{*}\right),\;\forall n\in N,a_{n}\neq a_{n}^{*} \end{array}$$

**Definition** **A2** (Exact Potential Game [35])**.** *Given a potential function φ, when a UAV changes its decision unilaterally, if the difference between the potential functions is equal to that between its utility functions, the game is an EPG with potential function φ. The following equation exists:*

(A2) $$\begin{array}{cc} u_{n}\left(a_{n},a_{-n}\right)-u_{n}\left(a_{n}^{\prime },a_{-n}\right)=\varphi \left(a_{n},a_{-n}\right)- & \varphi \left(a_{n}^{\prime },a_{-n}\right),\;\forall a_{n},a_{n}^{\prime }\in A \end{array}$$

In EPG, the change in the utility function is the same as the change in the potential function caused by any UAV changing its own strategy.

**Lemma** **A1.**
*The EPG has at least one pure Nash Equilibrium.*

**Proof.** We define the potential function φ as:

(A3) $$\begin{array}{c} \varphi \left(a_{n},a_{-n}\right)=\sum_{S_{k}\in \Pi }U_{S_{k}}\left(a_{n},a_{-n}\right) \end{array}$$

where $U_{S_{k}}\left(a_{n},a_{-n}\right)$ is the utility function of coalition $S_{k}$ when the decision of UAV *n* is $a_{n}$. It is seen that the potential function is the system utility of the network. When the coalition selection of UAV *n* changes from $a_{n}$ to $a_{n}^{\prime }$, the difference between the utility functions based on Cooperation order can be derived as:

(A4) $$\begin{array}{cc} \; & \;\;\;\;\;u_{n}\left(a_{n}^{\prime },a_{-n}\right)-u_{n}\left(a_{n},a_{-n}\right)\\ \; & =\sum_{k\in S_{i}}u_{k}\left(a_{n}^{\prime },a_{-n}\right)+\sum_{k\in S_{j}\setminus \left\{n\right\}}u_{k}\left(a_{n}^{\prime },a_{-n}\right)-\left[\sum_{k\in S_{i}\setminus \left\{n\right\}}u_{k}\left(a_{n},a_{-n}\right)+\sum_{k\in S_{j}}u_{k}\left(a_{n},a_{-n}\right)\right]\\ \; & =\left[U\left(S_{i}\right)+U\left(S_{j}\setminus \left\{n\right\}\right)\right]-\left[U\left(S_{i}\setminus \left\{n\right\}\right)+U\left(S_{j}\right)\right] \end{array}$$

where the coalition action of UAV *n* changes from $a_{n}$ to $a_{n}^{\prime }$, and UAV *n* leaves coalition $S_{j}$ and joins coalition $S_{i}$. □

For potential function, when the choice of UAV *n* changes from $a_{n}$ to $a_{n}^{\prime }$, the difference between two potential functions can be expressed as:

(A5) $$\begin{array}{cc} \; & \;\;\;\;\;\varphi \left(a_{n}^{\prime },a_{-n}\right)-\varphi \left(a_{n},a_{-n}\right)\\ \; & =\sum_{S_{i}\in \Pi }U_{S_{i}}\left(a_{n}^{\prime },a_{-n}\right)-\sum_{S_{j}\in \Pi }U_{S_{j}}\left(a_{n},a_{-n}\right)\\ \; & =\left[U\left(S_{i}\right)+U\left(S_{j}\setminus \left\{n\right\}\right)\right]-\left[U\left(S_{i}\setminus \left\{n\right\}\right)+U\left(S_{j}\right)\right]+\sum_{k\in \Pi \setminus \left\{S_{i},S_{j}\right\}}△U\left(S_{k}\right) \end{array}$$

where $△U\left(S_{k}\right)$ is the difference of utility functions for other coalitions. Since UAVs can only choose between the original and new coalition, no changes have been made to other coalitions. Therefore, the utility function for other coalitions is not related to the action that UAV *n* joins and leaves. Thus, $△U\left(S_{k}\right)=0$ and the difference between two potential functions can be simplified as:

(A6) $$\begin{array}{cc} \; & \;\;\;\;\;\varphi \left(a_{n}^{\prime },a_{-n}\right)-\varphi \left(a_{n},a_{-n}\right)\\ \; & =\left[U\left(S_{i}\right)+U\left(S_{j}\setminus \left\{n\right\}\right)\right]-\left[U\left(S_{i}\setminus \left\{n\right\}\right)+U\left(S_{j}\right)\right]\\ \; & =u_{n}\left(a_{n}^{\prime },a_{-n}\right)-u_{n}\left(a_{n},a_{-n}\right) \end{array}$$

It is seen that the difference between the utility functions due to the unilateral action of UAV *n* is the same with that between the potential functions. According to Definition A2, the game is an EPG, which has at least a pure NE. According to the definition of Nash Equilibrium, the UAVs cannot achieve better utility from unilaterally changing action, which also means that the stable coalition state is achieved. Therefore, Theorem 2 is proved.
